# Supplementary material for: Chitosan Oligosaccharide Supplementation Affects Immunity Markers in Ewes and Lambs during Gestation and Lactation
Source: Animals (Basel). 2022 Sep 28;12(19):2609. doi: 10.3390/ani12192609 (PMC9558557; doi:10.3390/ani12192609)
Supplement: Supplementary file 1 [file animals-12-02609-s001.zip › animals-1874335-supplementary.pdf]

Table S1. The nutrient and mineral content in the commercial loose lick.

	Nutrient and mineral break down		
	ANP - StockMins CLS		
Daily intake (kg/hd/day)	0.025		
	Macro analysis		
Paramater	%	g/hd/day	
Dry matter	97.39		
Mj ME/kg DM	0.48	0	Mj ME/day
CP	0.24	0	
NDF	0.02	0	
ADF	0.01		
NFC	0.00		
Fat	0.02	0	
Starch	0.01	0	
Sugar	2.52	1	
	Macro mineral analysis		
Mineral	%	g/hd/day	
Ash	61.01	15.3	
Ca	10.97	2.7	
P	0.00	0.0	
Mg	16.23	4.1	
K	0.16	0.0	
S	0.02	0.0	
Na	11.93	3.0	
Cl	18.28	4.6	
	Micro mineral analysis		
Mineral	mg/kg	mg/hd/day	
Co	0	0	
Cu	3	0	
I	0	0	
Fe	11	0	
Mn	2	0	
Se	0	0.00	
Zn	1	0	
Mo	0	0.00	
	Vitamins analysis		
Vitamin	IU/kg	IU/hd/day	
Vitamin A	0	0	
Vitamin D	0	0	
Vitamin E	0	0	
	mg/kg	mg/hd/day	
Vitamin B1	0	0	
Vitamin B6	0	0	
	Addatives		
	mg/kg	mg/hd/day	
Lasalocid	0	0	
DCAD	60	mEq/kg	
